# Supplementary material for: Hospital-wide natural language processing summarising the health data of 1 million patients
Source: PLOS Digit Health. 2023 May 9;2(5):e0000218. doi: 10.1371/journal.pdig.0000218 (PMC10168555; doi:10.1371/journal.pdig.0000218)
Supplement: Attachment — Submitted filename: PLOS DH _ hospital wide - response to reviewers.docx [file pdig.0000218.s001.docx]

Journal requirements

1. Please review your reference list to ensure that it is complete and correct. If you have cited papers that have been retracted, please include the rationale for doing so in the manuscript text, or remove these references and replace them with relevant current references. Any changes to the reference list should be mentioned in the rebuttal letter that accompanies your revised manuscript. If you need to cite a retracted article, indicate the article's retracted status in the References list and also include a citation and full reference for the retraction notice.

We have updated our reference list to cite URLS rather than including them in the main text and have updated the format to PLOS requirements.

2. Please send a completed 'Competing Interests' statement, including any COIs declared by your co-authors. If you have no competing interests to declare, please state "The authors have declared that no competing interests exist". Otherwise please declare all competing interests beginning with the statement "I have read the journal's policy and the authors of this manuscript have the following competing interests:"

We have added the competing interests statement

3. Thank you for including an Ethics Statement for your study. Please include a statement that formal consent was obtained (must state whether verbal/written) OR the reason consent was not obtained (e.g. anonymity). NOTE: If child participants, the statement must declare that formal consent was obtained from the parent/guardian.

We have updated the ethics statement.

4. Please provide your detailed Financial Disclosure statement. This is published with the article. It must therefore be completed in full sentences and contain the exact wording you wish to be published.

We have added the financial disclosure statement

5. We ask that a manuscript source file is provided at Revision. Please upload your manuscript file as a .doc, .docx, .rtf or .tex.

We have provided the source file.

Reviewer #1: The submitted article is well organized and written. The topic is quite relevant and demonstrates how health-care institutions can leverage their unstructured data to extract knowledge that can help stakeholders to make crucial decisions. This article is also relevant by the number of patients/medical notes included in this work (1.07 M patients).

The main comments that I have for authors are:

- You were able to summarize data and extract valuable information and made a good analysis grouping by gender. Also, you stated very well your limitations. However, you should add a comment regarding ethnicities and disparities. For instance, can it be generalized to all ethnicities and minorities in your dataset?

We have added a comment to the discussion regarding ethnicity. While we have a large patient sample from a diverse area we do not claim the results will be generalisable to the wider population or to specific ethnicities or other backgrounds.

- Your sample used for clustering how well describes your population. Does demographics are distributed equally compared to the full dataset? Any biased identified during selection?

The sample is not significantly different to the remaining patients in terms of age (t-test p=0.45) or sex distribution (fisher exact test p=0.90). We have added this detail to the manuscript.

- From my opinion and as Data Scientist, its biggest pain point is that I or anyone will not be able to reproduce your work in other datasets. You are not allowed to share dataset according to your Data Availability Statement. Nonetheless, you should be able to share your code for guidance. This would help enormously the scientific community for future work downstream. You can also share the framework that you used for reaching these results (e.g., training pipelines, pre-processing pipelines, etc.)

We have added the clustering script to the public github repository that contains the clustering results, available at: <https://github.com/KHP-Informatics/NLP-embedding-visualisation/blob/main/clustering.ipynb>

- I hoped to see performance metrics for your clustering algorithms. For example, which metrics did you used to select the number of clusters?

As stated in the Methods section we analysed cluster inertia to determine the appropriate number (the “elbow method”). As stated in the Results, we do not consider this to be a globally optimal solution to patient clustering and we use the clusters largely to sample regions of the embedding space.

- Another important aspect that could bring more impact to scientific community is sharing advices and suggestions when you are working with medical notes that are not written in English. Do the tools you used for this work support multilingual inputs?

We agree that medical NLP in languages other than English is an important topic. However we have not handled any non-english text in our paper and we have not applied any of our tools to any non-english text. We therefore do not have any advice to offer the community on this topic. The MedCAT pipeline is built on spacy and could be trained for use with other languages than english. Anecdotally we are aware of collaborators using MedCAT with Danish language medical notes. However as we do not make any contribution to non-english text ourselves and do not have public results for our collaborators we have not changed the manuscript in this case.

- Why did not you used python libraries widely used for NLP tasks? (e.g., Spacy, NLTK, etc.). The MedCAT NLP library performs better?

The MedCAT library is based on standard tools (spacy and huggingface). Those tools do not provide the trained models needed for medical NER+L performed in our work. We used MedCAT in this work because we have previously trained models on our data using MedCAT, as cited in our methods.

- How did you pre-process your free text? You did not provide enough detail for future reproduction in other datasets.

We have provided further detail on our MedCAT configuration and document preprocessing.

Reviewer #2: -

The URL in the second paragraph of the Results section in page 3 could be set as a citation in the end of the document

We have replaced all in-text urls with citations.

- Figure 2 is hard to read and the authors would benefit by deleting "(disorder)" from the labels in the Z axis

We have updated figure 2 accordingly.

- Following the reference to Table 2 in page 5 the authors could add "presented in the appendix of this paper"

We apologise for the confusion, Table 2 is not an appendix and will appear in the main text in the final published version. All tables are at the end of the document in the submitted version.

- The disease acronyms provided in the caption of Figure 3 could be provided earlier in the body of the text, as they are mentioned before the Figure is exposed.

Where disease acronyms used in figure 3 are also used in the main text we have now added the definition to the text.

- In the paragraph following Figure 3, please specify which clustering algorithm was used and why

We have added the name of the clustering algorithm to the text (agglomerative clustering). However we feel that further detail on the method should remain in the Methods section where we have also added more detail.

- In the paragraph following Figure 3, please substitute the URL by a standard citation described in the end of the paper. Similarly to the URL appearing in the caption of Figure 4

We have replaced all in-text urls with citations.

- I would like to have read the contribution of the paper earlier in the paper (at least summarised in the Introduction section), from the perspective of the last two paragraphs of page 9 and its relation with existing work

We have expanded briefly on the contribution of our work in context at the end of the introduction as we feel it is covered sufficiently in the discussion.
